# Evaluation of three formulations based on Polymorphic membrane protein D in mice infected with *Chlamydia trachomatis*

**DOI:** 10.3389/fimmu.2023.1267684

**Published:** 2023-11-16

**Authors:** Romina Cecilia Russi, Diego del Balzo, Ivana Gabriela Reidel, Mariano Alonso Bivou, Noelia Flor, Agustín Lujan, Diego Sanchez, María Teresa Damiani, Carolina Veaute

**Affiliations:** ^1^Laboratorio de Bioquímica e Inmunidad, Facultad de Ciencias Médicas, Universidad Nacional de Cuyo, Instituto de Medicina y Biología Experimental de Cuyo, Consejo Nacional de Investigaciones Científicas y Técnicas (IMBECUCONICET), Universidad Nacional de Cuyo, Mendoza, Argentina; ^2^Experimental Immunology Laboratory, School of Biochemistry and Biological Sciences, National University of Litoral, Ciudad Universitaria, Santa Fe, Argentina; ^3^Department of Immuno-Oncology, Beckman Research Institute of City of Hope, Duarte, CA, United States

**Keywords:** PmpD, *Chlamydia trachomatis*, vaccine, adjuvants, CpG, gemini

## Abstract

The significant impact of *Chlamydia trachomatis(Ct)* infections worldwide highlights the need to develop a prophylactic vaccine that elicits effective immunity and protects the host from the immunopathological effects of *Ct* infection. The aim of this study was to evaluate a vaccine based on a fragment of the Polymorphic membrane protein D (FPmpD) of *C. trachomatis* as an immunogen using a heterologous DNA prime-protein boost strategy in female mice Three different formulations were evaluated as protein boost: free recombinant FPmpD (rFPmpD) or rFPmpD formulated with a liposomal adjuvant alternatively supplemented with CpG or a cationic *gemini* lipopeptide as immunostimulants. The three candidates induced an increase in the cervicovaginal and systemic titers of anti-rFPmpD antibodies in two strains of mice (BALB/c and C57BL/6), with no evidence of fertility alterations. The three formulations induced a rapid and robust humoral immune response upon the *Ct* challenge. However, the booster with free rFPmpD more efficiently reduced the shedding of infective *Ct* and prevented the development of immunopathology. The formulations containing adjuvant induced a strong inflammatory reaction in the uterine tissue. Hence, the prime-boost strategy with the adjuvant-free FPmpD vaccine formulation might constitute a promissory candidate to prevent *C. trachomatis* intravaginal infection.

## 1 Introduction

*Chlamydia trachomatis (Ct)* is a bacterium that produces acute and chronic diseases with a significant impact on public health (1, 2). Chlamydial disease pathogenesis is considered to be mainly immune-mediated, with exacerbated mucosal inflammatory reactions. Inflammatory responses are frequently triggered by asymptomatic infections capable of spreading throughout the genital tract. This spread causes tissue deterioration and scarring, precipitating a spectrum of complications, including infertility in women (3). On the other hand, urethritis and inflammation of the accessory glands are the more common complications in men (1). In the year 2012, the World Health Organization (WHO) conducted an estimation, revealing staggering 131 million incidents of newly emerging chlamydial infections within the global population of adults and adolescents, specifically those aged between 15 and 49 years (4). The significant impact of *Ct* worldwide highlights the need to develop more efficient preventive tools (3, 5). To date, no successful prophylactic vaccine against *Ct* has been developed, in part because the pathogenesis of *Ct* and the function of the host immune response has not been fully elucidated. Several studies suggested that protective immunity against *Ct* is associated with a Th1 response and neutralizing antibodies (6). Furthermore, considering the recurrence of *Ct* re-infections and the persistence of chronic infections, it becomes evident that the establishment of a robust, enduring anti-chlamydial immune memory response is imperative (7). While the relevance of mucosal immune response generation for bacterial clearance is evident (7), it might be associated with tissue damage and pathologic sequelae (8). Therefore, a successful vaccine design involves a delicate balance between triggering a sufficiently robust protective immune response and the potential immune-pathological damage associated with the host response.

Adjuvants have been widely used to improve vaccine effectiveness. The precise and appropriate combination of antigens and adjuvants is of paramount importance in the formulation of an effective vaccine. Most new-generation adjuvants included in licensed vaccines or clinical trials consist of a delivery system supplemented with an immunostimulant (9). This combination can improve vaccine efficacy in different ways; increasing the immune response to poorly immunogenic antigens, inducing a specific T cell profile, and reducing the antigen dose or the number of boosters needed to achieve proper protection (10). Therefore, the selection of both the delivery system and the immunostimulants capable of inducing an adequate immune response to control the pathogen infection is crucial. Liposomes, as phospholipid vesicles, are known for their remarkable ability to promote targeted immune responses, carrying antigens together with immunostimulatory molecules (11–13). The immunostimulatory potential of synthetic oligodeoxynucleotides containing unmethylated CpG motifs (ODN-CpG) has been subjected to comprehensive evaluation. Previous studies have convincingly demonstrated that the incorporation of ODN-CpG into diverse vaccine formulations, evaluated in preclinical and clinical trials, triggers the activation of professional antigen-presenting cells. This activation enhances the humoral and cellular immune responses, as well as their duration (14, 15). Moreover, previous work demonstrated that the combination of liposomes with ODN-CpG provides better results than using the individual components and leads to a Th1 cellular immune response profile (16, 17). Another group of nanoparticles applied in nanotechnology that have attracted great interest as agents for drug delivery are the *gemini* lipopeptides (18). These lipopeptides comprise a hydrophobic chain of natural fatty acids (normally between 12 and 18 carbons) and a polar region constituted by an oligopeptide. In particular, those that include cysteine in their structure can form dimers in solution at physiological pH from monomeric structures, becoming *gemini* and giving rise to the formation of stable supramolecular systems capable of encapsulating DNA or RNA, proteins, and/or drugs for therapeutic use (19–21). Our group designed and synthesized a family of new cysteine-containing lipopeptides. During the characterization of these molecules, some of them demonstrated adjuvant capacity, such as the AG2-C16 molecule (22).

Several authors agree that further work is necessary to identify new immunogen candidates and delivery mechanisms that meet the criteria of safety, and immunogenicity with effective induction of protective immunity (23). In addition, diverse studies highlight Polymorphic membrane protein D (PmpD) as a potential candidate as it can induce pan-serovar neutralizing antibodies, which would be essential to a broadly effective vaccine (23, 24). Previously, we designed an immunization strategy based on a selected fragment of PmpD and a heterologous regimen with DNA (prime) followed by two boosts of the recombinant protein (25). Our study conclusively exhibited that this vaccine elicits an immune response effective against intravaginal *Ct* infection. In this work, we hypothesized that the formulation of the protein with new adjuvants would provide better performance to the vaccine.

Therefore, we designed and evaluated three different formulations as protein boost; free recombinant FPmpD (rFPmpD) or rFPmpD formulated with a liposomal adjuvant alternatively supplemented with ODN-CpG or a cationic *gemini* lipopeptide. We then used a murine model of female genital *Ct* infection, to analyze the protective efficacy of the three vaccine formulations, and their impact on infection-related immunopathology. The results of our investigation suggest that while the vaccine with free rFPmpD elicited a protective immune response, the vaccine formulations with adjuvants did not show further protection against infection and infection-induced immunopathological damage.

## 2 Materials and methods

### 2.1 Cell culture and *Chlamydia trachomatis* strains

*Chlamydiatrachomatis* serovar L2 434/Bu (Ct) was kindly provided and characterized by the Unidad de Estudios de Clamidias, UBA, Buenos Aires, Argentina. Serovar D was generously contributed by the Instituto Malbran, Buenos Aires, Argentina. Additionally, a fluorescent *Ct* L2 strain with green fluorescence, containing the p2TK2-SW2IncDProm-RSGFP-IncDTerm serovar L2 construct (GFP-*Ct*), was kindly supplied by Dr. Derré (26). *Ct* bacteria were cultivated within HeLa cells (ABAC, Argentina) using high glucose Dulbecco’s Modified Eagle’s Medium (GIBCO, Thermo Fisher Scientific, USA) supplemented with 10% (v/v) fetal bovine serum (FBS) sourced from Internegocios SA, Argentina. The growth medium was further enriched with 1.55 mg/mL glucose (Biopack, Argentina) and 0.3 mg/mL L-glutamine (ICN Biomedicals Inc, USA), and antibiotics were omitted. Cultures were maintained in a 5% CO2 environment at 37°C. Chlamydial elementary bodies (EBs) were harvested, purified, and quantified following the methodology previously outlined by Del Balzo et al. (27).

### 2.2 Preparation of vaccine formulations

The design, cloning, and purification of plasmid construction pVAX1−FPmpD for DNA immunization and the production of recombinant PmpD protein fragment (rFPmpD) for boosters were previously described (25). Cationic liposomes (Lp) were prepared by the ethanol injection method using 4 mM as the final lipid concentration in 7:2:2 mol/mol ratio of DPPC: Chol:SA. The ODN-CpG (CpG) with the sequence: 5’-tcgtcgtttgtcgttttgtcgtt-3’ was obtained by chemical synthesis (Invitrogen, USA), with phosphodiester bonds (17). The *gemini* lipopeptide AG2-C16 (Gem) was synthesized using 9-fluorenyl-methoxycarbonyl (Fmoc) chemistry in the solid phase, according to Grippo et al., 2019 (22). The purified DNA was resuspended in sterile sodium acetate buffer (0.05 M; pH 5.3) at 2.5 μg/μL. The protein formulations were prepared alternatively as: free rFPmpD, rFPmpD+Lp+CpG, rFPmpD+Lp+Gem, or rFPmpD+Lp, in sodium acetate buffer (0.05M; pH 5.3). Final concentrations were: 7.5 nmol/mL CpG, 400 μM Gem, 0,125 μg/μL (intranasal route) and 0,05 μg/μL (subcutaneous route) rPmpD.

### 2.3 Mice

Female BALB/c mice (Icivet Litoral, Argentina) and female C57BL/6 mice (The Jackson Laboratory, USA), aged six to eight weeks, were housed in a controlled environment that met specific pathogen-free (SPF) criteria. The housing conditions included a humidity level of 50-60%, a temperature range of 20-24°C, 60 air exchanges per hour within the cages, and a 12/12 hour light/dark cycle with lights turning on at 8 am. The mice were provided with *ad libitum* access to drinking water and a standardized mouse diet. All experimental procedures were conducted in accordance with the guidelines outlined in the Guide for the Care and Use of Laboratory Animals (ILAR 2010). Animal protocols were granted approval by both the Institutional Advisory Committee on Research Ethics and Security (FBCB-UNL, Argentina) and the Institutional Committee on Laboratory Animal Care and Use (CICUAL, UNCUYO, Argentina). This study adheres to the standards set by the ARRIVE guidelines (https://arriveguidelines.org).

### 2.4 Vaccination scheme

**Figure 1:**
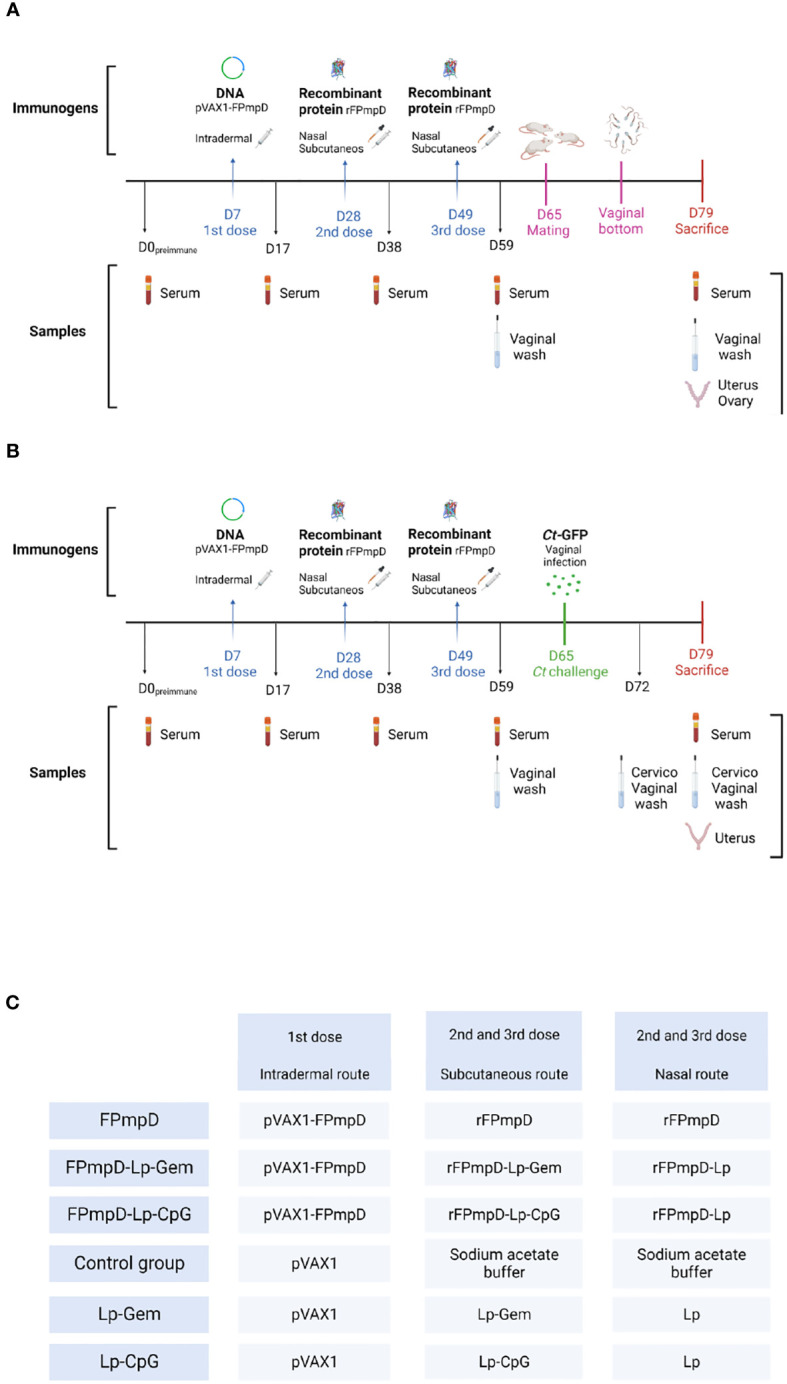
Temporal Framework illustrating the experimental protocol for two strains of female mice: BALB/c **(A)** and C57BL/6 **(B)**. A diagrammatic depiction of the immunogen and administration route employed for immunization, and the type of sample collected at every temporal instance. The experimental day is indicated with the letter D. **(C)** For the two mice strains, immunogens were rFPmpD alone; rFPmpD + liposomes (Lp) + ODN-CpG (CpG); rFpmpD + liposomes (Lp) + gemini lipopeptide AG2-C16 (Gem) according to the experimental groups: rFPmpD; rFPmpD-Lp-CpG; rFPmpDLp- Gem, respectively. Control groups were inoculated with the empty plasmid (Control) or the both adjuvants without the recombinant protein (Lp- CpG and Lp-Gem).

Animals were randomly assigned to one of these groups: FPmpD, FPmpD-Lp-CpG or FPmpD-Lip-Gem (BALB/c and C57BL/6; n=6 and n=5 per group, respectively). A DNA prime-protein boost strategy was applied, with a total of three doses, three weeks apart. Mice from the three groups received an ear-pinnae intradermal first dose of 50 μg FPmpD-pVAX1. Two protein boosts were administered by the intranasal (2.5 μg) and subcutaneous (5 μg) routes, simultaneously. For the intranasal immunization free rFPmpD was administered to the FPmpD group, and rFPmpD+Lp was used for the groups FPmpD-Lp-CpG and FPmpD-Lp-Gem. For the subcutaneous injections, free rFPmpD, rFPmpD+Lp+CpG or rFPmpD+Lp+Gem were administered. The adjuvant control groups (BALB/c and C57BL/6; n=5 and n = 3, respectively) received empty plasmid (pVAX1) in the first dose followed by two doses of the adjuvants, without protein (Lp-Gem or Lp-CpG). Two additional control groups (BALB/c and C57BL/6; n = 5 and n = 4, respectively) were inoculated with pVAX1 in the first dose, and acetate buffer in the second and third doses (Control). Blood was collected from the submandibular vein seven days (d) before immunization and ten days after each inoculation and sera were obtained and stored at − 20°C until use. Vaginal fluid (VF) was collected by vaginal washing using PBS ten days after the last dose, with the addition of protease inhibitors cocktail Set I (Merck, Germany). The collected samples were stored at − 80°C until use. Upon sacrifice, which occurred 30 d post last dose, mice were subjected to cardiac puncture for blood collection, under anesthesia. Anesthesia was induced using 100 mg/kg Ketamine (Hollyday-Scott SA, Argentina) and 10 mg/kg Xylazine (König SA, Argentina). The sera were stored at -20°C until use. Timelines that represent experimental protocols and the experimental groups with the vaccinal formulations administered are displayed in **Figure 1**.

### 2.5 Measurement of anti−PmpD antibodies in serum and vaginal fluid

Specific antibodies against rFPmpD was measured by an indirect enzyme-linked immunosorbent assay (ELISA) both in sera and VF samples. In brief, 96-well polystyrene plates (Microlon™ 600, Greiner Bio-One, Austria) were coated overnight at 4°C with rFPmpD (1 μg/100μL) diluted in carbonate buffer. Following this, non-specific binding sites were blocked using PBS supplemented with 5% skimmed milk. Sera were diluted 1:100, and VF was diluted 1:3 in PBS-1%-skimmed milk. The plates were incubated for 1 hour at 37°C with the respective peroxidase-conjugated anti-mouse antibodies: anti-mouse IgG (Jackson, USA), anti-mouse IgG1 (Santa Cruz Biotechnology, Inc., USA), anti- mouse IgG2a (Abcam Inc., USA), anti-mouse IgG2c (Abcam Inc., USA), or anti-mouse IgA (Sigma-Aldrich, USA), as appropriate. Plates were washed 3 times after each step using PBS-0.05% Tween 20. Finally, the plates were incubated with the substrate (hydrogen peroxide) and the chromogen tetramethylbenzidine (Thermo Scientific, USA). The reaction was stopped by adding 0.5 M H_2_SO_4_. The Optical Density (OD) was read at 450 nm using a microplate reader (Mulstiskan EX, Labsystems, USA). The obtained results are presented in terms of OD. The antibody levels in serum samples collected 30 d post-last inoculation from FPmpD-vaccinated (FPmpD, FPmpD-Lp-Gem and FPmpD-Lp-CpG), and non-vaccinated mice (Control, Lp-CpG, Lp-Gem) were quantified by titration. The cut-off value was determined by calculating the mean OD of the control group and adding three times the standard deviation.

### 2.6 Fertility assessment of female mice

Female BALB/c mice from all experimental groups were mated with untreated male mice aged 14–16 weeks with proven fertility. Each cage contained three female mice paired with a single male mouse. Mating took place after a ten-day period following the last administered dose. The presence of vaginal plugs or spermatozoa in the vaginal secretions was monitored daily, and the day of detection marked the beginning of pregnancy (designated as day 0). The female mice were then separated and kept until day 18 of pregnancy, when they were sacrificed. Uteri and ovaries were collected, and various reproductive parameters were recorded, including the counts of corpora lutea, implantations, resorptions, as well as both live and dead fetuses. Subsequently, the following parameters were calculated: Fertility potential (efficiency of implantation): Calculated as (number of implantation sites/number of corpora lutea) × 100. Rate of preimplantation loss: Calculated as [(number of corpora lutea - number of implantations)/number of corpora lutea] × 100. Rate of postimplantation loss: Calculated as [(number of implantations - number of live fetuses)/number of implantations] × 100, following the methodology described by Perobelli et al. (28).

### 2.7 Intravaginal *Ct* challenge infection and bacterial burden

The intravaginal infection mouse model was performed according to Lujan et al. (6). On day 16 after the last dose, C57BL/6 mice of all the experimental groups were intravaginally challenged using 1.5 × 10^5^ GFP-*Ct* serovar L2 EBs (6). *Ct* serovar L2 was employed as described in Shaw et al. (29, 30). To synchronize the estrous cycle of the C57BL/6, a single subcutaneous injection of 2.5 mg of medroxyprogesterone acetate (Holliday-Scott SA, Argentina) were administered seven d prior to the GFP-*Ct* infection. Cervicovaginal swabs were collected at seven- and 14 d post-challenge from each experimental group using disposable microapplicators (Multi-Brush, Denbur Inc, USA). These swabs were then cultured on a monolayer of HeLa cells. The quantification of infectious bacterial progeny present in the cervicovaginal secretions was performed by assessing Inclusion Forming Units (IFU) using confocal microscopy and flow cytometry, according to Vromman et al. (7). The reduction in IFU upon vaccination was considered protection. Protection was examined in swab samples on day seven based on previous data (25, 31, 32).

### 2.8 Uterine horns histology and morphology

On the 14th day after infection (14 d p.i.), the mice were euthanized, and their uterine horns were dissected for both morphological and histological examination. The length of the uterine horns was quantified. Subsequently, one uterine horn from each mouse was fixed in a solution of 4% paraformaldehyde and then subjected to embedding in paraffin using standard protocols. Thin tissue slices, 5 μm in thickness, were prepared and stained using hematoxylin and eosin. The stained slices were observed under a Leica DMi8 microscope, which was equipped with an MShot color camera, facilitating detailed histological analysis. The resulting images were further processed using M-Shot analysis system software v 1.1.6 (Micro-Shot, China).

### 2.9 Immunohistochemistry

The uterine sections (5 μm thick) were immunostained for the detection of macrophages and the expression of TNFα. At 4°C, the samples were overnight incubated with primary antibodies: anti-CD68, 1:200 (Invitrogen, USA); anti-TNFα, 1:50 (Invitrogen, USA). Then, the sections were incubated 1 h at room temperature with biotinylated anti-mouse (Vectastain Elite ABC universal kit, Vector Laboratories, USA) and 1:5.000 biotinylated anti-rat (Vector Laboratories, USA), respectively. Biotinylated antibodies were detected with Vectastain Elite ABC universal Kit, peroxidase (Vector Laboratories, USA), and finally, the reactions were developed using an ImmPACT DAB Peroxidase Substrate (Vector Laboratories, USA).

### 2.10 RNA extraction and RT-qPCR

Total RNA was isolated from a single uterine horn using TRIzol® Reagent (Invitrogen, USA). The extracted RNA was quantified by measuring OD at 260 nm employing a NanoDrop Lite spectrophotometer (Thermo Scientific, USA). Subsequently, one microgram of total RNA from each sample was retrotranscribed to cDNA using MMLV reverse transcriptase (Promega, USA). Quantitative Real-Time PCR was employed to determine the relative expression levels of TNFα, IL-1, IL-6, IFNγ, IL-10 mRNA. Specific primers were used for each target gene (as outlined in **Table 1**). Each qPCR was performed using HOT FIREPol® EvaGreen® qPCR Mix Plus (Solis Biodyne, Estonia) and a primer concentration of 0.5 μM for each primer. PCR amplifications were performed on a Corbett Rotor Gene 6000 Real-Time Thermocycler (Corbett Research Pty Ltd Sydney, Australia). The amplification protocol initiated with a denaturation step (12 min at 95°C), followed by 40 cycles of denaturation (20 sec at 95°C), annealing (30 sec at the temperature specified for each primer pair in **Table 1**), and extension (20 sec at 72°C). To ensure specificity, a melting curve analysis was routinely conducted for each reaction. The relative gene expression was calculated using the 2-DDCT method p and GAPDH as a housekeeping gene for normalization of the relative mRNA levels.

**Table 1:** Primer sequence used for qPCR. Ta is the annealing temperature for each pair of primers.

Primers	Forward	Reverse	Ta (°C)
mTNFα	gaccctcacactcagatcatcttct	Acgctggctcagccactc	55
mIL-1β	gaaagacggcacacccacc	Aaaccgcttttccatcttcttct	55
mIL-6	atccagttgccttcttgggactga	taagcctccgacttgtgaagtggt	60
mIL-10	gctcctaagagagttgtgaagaaactc	Agctgctgcaggaatgatca	55
mIFNγ	Atgaacgctacacactgcat	Taggctttcaatgactgtgc	55
mGAPDH	Tgcgacttcaacagcaactc	Cttgctcagtgtccttgctg	60

### 2.11 Statistical analysis

The results are presented as the mean ± standard error of the mean (SEM). The normality of the distribution was evaluated using the Kolmogorov-Smirnov test. Statistical differences among groups were assessed through appropriate methods, including two-tailed t-test or one-way and two-way ANOVA or Kruskal-Wallis, with multiple comparisons, using GraphPad Prism 8.0.1 software (GraphPad, USA). Statistical significance was determined with a threshold of p values less than 0.05.

## 3 Results

### 3.1 The three vaccine formulations elicited systemic and cervicovaginal humoral immune response against rPMPD in two mouse strains

**Figure 2:**
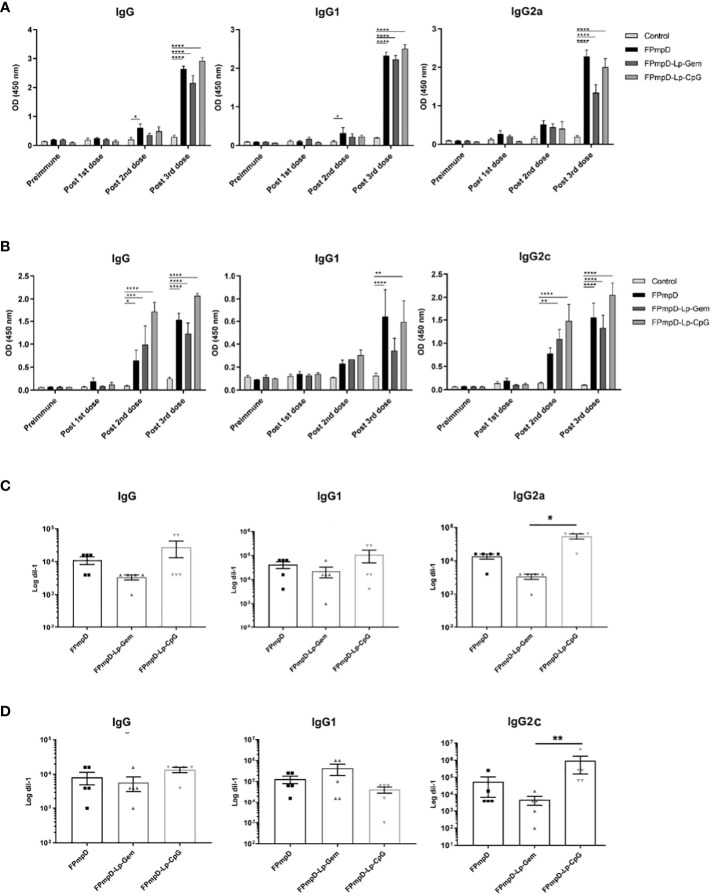
Systemic humoral immune response. Figures **(A, B)** illustrate the levels of anti-PmpD IgG, IgG1, IgG2a/c antibodies in sera obtained from both Control and FPmpD-vaccinated BALB/c **(A)** and C57BL/6 **(B)** mice, ten d after each dose. The bars withing the graphs depict the mean ± SEM for each experimental group, employing a dilution of 1:100. Statistical analysis was conducted using a two-way ANOVA with Sidak’s multiple comparisons test; revealing statistical significance denoted as *p<0.05, **p<0.01; ***p<0.001, ****p<0.0001). In figures C and D, the specific anti-PmpD IgG, IgG1 and, IgG2a/c titration in BALB/c **(C)** and C57BL/6 **(D)** murine sera, acquired ten d post the final administered dose. The bars in the plot indicate mean ± SEM. (Kruskal-Wallis with Dunn’s multiple comparisons test).

**Figure 3:**
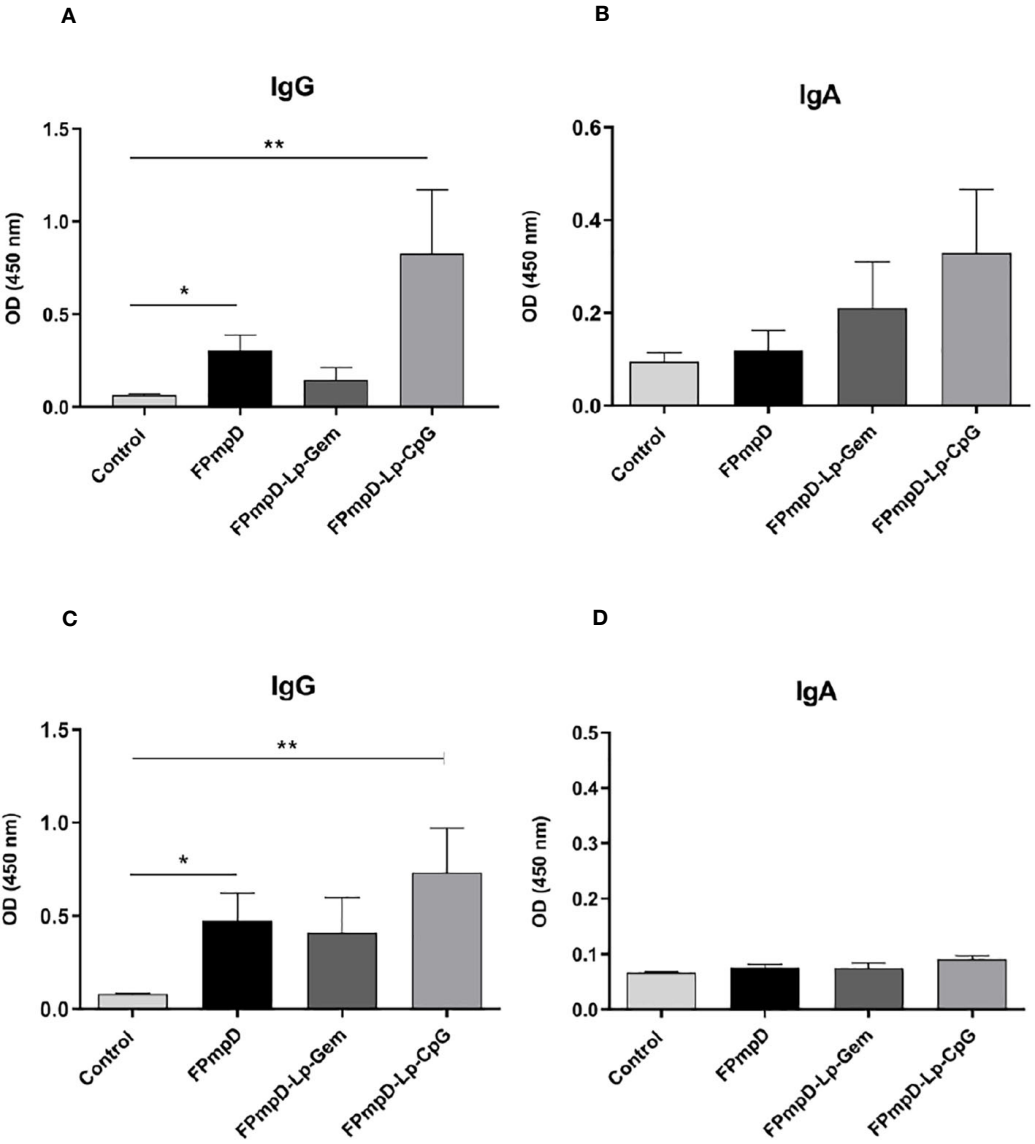
Mucosal humoral immune response. Figure **(A, B)** depict the levels of Anti-PmpD IgG and IgA antibodies in vaginal washes acquired ten d following the last dose from non-vaccinated (Control) and FPmpD-vaccinated BALB/c. In figures **(C, D)**, the same antibody levels are presented for FPmpD-vaccinated C57BL/6 mice. Each experimental group is denoted by bars representing the mean ± SEM for each experimental group. The analyses were conducted using a dilution of 1:3. Statistical significance is indicated as *p<0.05; **p<0.01, employing the Kruskal-Wallis with Dunn’s multiple comparison test).

The systemic humoral immune response was analyzed by measuring the anti-PmpDIgG, IgG1, and IgG2a/c serum levels before immunization (pre-immune), and ten days after each dose in BALB/c (**Figures 2A, C**) and C57BL/6 (**Figures 2B, D**) mice as indicated in Methods (**Figure 1**). The prime-boost regimen elicited demonstrable increments of anti-PmpD antibodies with the successive doses that became significant compared to the control after the second dose for IgG and IgG1 in BALB/c mice vaccinated with FPmpD (p<0.05), for IgG in the three groups of vaccinated C57BL/6 mice, and for IgG2c in the FPmpD-Lp-Gem and FPmpD-Lp-CpG groups of C57BL/6 mice vaccinated with adjuvants. After the third dose, all the vaccine formulations induced a significant further increase in the antibody levels, except for IgG1 in the FPmpD-Lp-Gem group of C57BL/6 mice (**Figure 2**). Specific anti-PmpD IgG, IgG1, or IgG2a/c antibodies were absent in the pre-immune sera of the experimental groups, as well as in the groups that received adjuvants without protein (Lp-Gem and Lp-CpG) during the entire trial period (**Table 2A**). The titers of IgG and the subclasses (IgG1 and IgG2a/c) anti-PmpD antibodies ten days after the last dose in the groups that received free rFPmpD or rFPmpD with adjuvants were between 10^4^ and 10^5^ dils^-1^ in animals of both strains (**Figures 2C, D**). FPmpD-Lp-CpG elicited higher IgG2 titers than FPmpD-Lp-Gem in both strains. However, none of the adjuvant groups showed a significant difference compared to the FPmpD group. We then proceeded to examine the efficacy of the FPmpD formulations in enhancing humoral immunity within the mucosal environment of the genital tract. To assess this, we quantified anti-PmpD IgA and IgG antibodies in vaginal wash samples collected ten days following the last immunization. We observed significantly higher levels of IgG in vaginal washes from mice vaccinated with free FPmpD (FPmpD; p<0.05) and with FPmpD-Lp-CpG (p<0.01) in BALB/c (**Figure 3A**) and C57BL/6 (**Figure 3C**) mice, compared to control ones. While the FPmpD formulations, both with and without adjuvants, did induce specific anti-PmpD IgA antibodies in the mucosal genital tract of certain BALB/c mice, the mean IgA levels observed did not exhibit significant differences when compared to the non-vaccinated control mice (as shown in **Figures 3B, D**). Pre-immune vaginal washes and the Lp-Gem and Lp-CpG groups displayed no presence of specific IgG or IgA throughout the duration of the study (as outlined in **Table 2B**). These results collectively indicate that FPmpD elicits a robust systemic and mucosal IgG humoral immune response, while the inclusion of any of the tested adjuvants does not seem to enhance this response.

Table 2Levels of anti-PmpD antibodies in Lp-Gem and Lp-CpG groups expressed as Mean ± SEM in sera (A) and in vaginal fluid (B).

**Table:** 

A						
	Lp-Gem			Lp-CpG		
BALB/c	IgG	IgG_1_	IgG_2a_	IgG	IgG_1_	IgG_2a_
Pre-immune	0.141 ± 0.01	0.095 ± 0.008	0.095 ± 0.008	0.187 ± 0.028	0.083 ± 0.008	0.083 ± 0.008
Post 1^st^ dose	0.187 ± 0.062	0.110 ± 0.028	0.123 ± 0.032	0.142 ± 0.02	0.073 ± 0.004	0.2 ± 0.056
Post 2^nd^ dose	0.204 ± 0.067	0.104 ± 0.023	0.154 ± 0.044	0.251 ± 0.049	0.098 ± 0.009	0.214 ± 0.063
Post 3^rd^ dose	0.287 ± 0.073	0.197 ± 0.008	0.190 ± 0.037	0.215 ± 0.053	0.236 ± 0.043	0.263 ± 0.062
	Lp-Gem			Lp-CpG		
C57BL/6	IgG	IgG_1_	IgG_2c_	IgG	IgG_1_	IgG_2c_
Pre-immune	0.067 ± 0.002	0.105 ± 0.003	0.091 ± 0.009	0.068 ± 0.003	0.110 ± 0.002	0.104 ± 0.009
Post 1^st^ dose	0.083 ± 0.018	0.135 ± 0.026	0.151 ± 0.045	0.117 ± 0.019	0.112 ± 0.002	0.104 ± 0.013
Post 2^nd^ dose	0.123 ± 0.026	0.142 ± 0.008	0.153 ± 0.003	0.166 ± 0.042	0.119 ± 0.014	0.171 ± 0.061
Post 3^rd^ dose	0.277 ± 0.033	0.136 ± 0.027	0.162 ± 0.048	0.281 ± 0.024	0.128 ± 0.015	0.123 ± 0.02

**Table:** 

B				
	Lp-Gem		Lp-CpG	
BALB/c	IgG	IgA	IgG	IgA
Post 3^rd^ dose	0.063 ± 0.006	0.061 ± 0.006	0.067 ± 0.011	0.127 ± 0.042
C57BL/6	IgG	IgA	IgG	IgA
Post 3^rd^ dose	0.069 ± 0.001	0.071 ± 0.001	0.085 ± 0.007	0.072 ± 0.002

### 3.2 Vaccine formulations based on PmpD did not alter mice’s fertility

We conducted an assessment of the potential impact of vaccination on mice fertility considering that an intensified immune response in the mucosal region might negatively influence the functioning of reproductive organs (33). The fertility potential, evaluated by implantation efficiency, remained unaltered in vaccinated mice when compared to the non-vaccinated control group (Kruskal-Wallis test; p > 0.05; **Table 3**). Furthermore, we computed the pre-implantation loss rate by counting the number of corpora lutea and implantations, and the post-implantation loss rate by considering the number of implantations and live fetuses. Our analysis revealed no notable distinctions in either the pre-implantation or post-implantation loss rates between the vaccinated and non-vaccinated mice. This suggests that the FPmpD prime-boost strategy did not hinder fertility (Kruskal-Wallis test; p > 0.05; **Table 3**). Moreover, there were no significant differences in the fertility parameters between any of the adjuvant control groups (Lp-Gem, Lp-CpG) and the non-vaccinated mice (Kruskal-Wallis test; p>0.05). In summary, the scheme of immunization with the vaccine formulations assayed in this study does not alter fertility.

**Table 3:** Fertility Parameters. Rate of pre-implantation loss, post-implantation loss, and fertility potential.

Fertility Parameters	Control	FPmpD	Lp-Gem	FPmpD-Lp-Gem	Lp-CpG	FPmpD-Lp-CpG
**Pre-implantation loss (%)**	32.1 ± 12.3	34.1 ± 6.9	29.2 ± 15.4	41.3 ± 11.7	57.1 ± 14.9	41.4 ± 6.6
**Post-implantation loss (%)**	15.9 ± 9.5	25.5 ± 8.8	11.9 ± 7.8	8.5 ± 5.1	33.3 ± 23.5	26.1 ± 14.4
**Fertility potential (%)**	49.2 ± 7.8	53.8 ± 8.4	51,9 ± 11.1	44,2 ± 8.3	38.4 ± 17.4	46.1 ± 4.4

### 3.3 FPmpD vaccine without adjuvants reduces the shedding of infective bacteria

**Figure 4:**
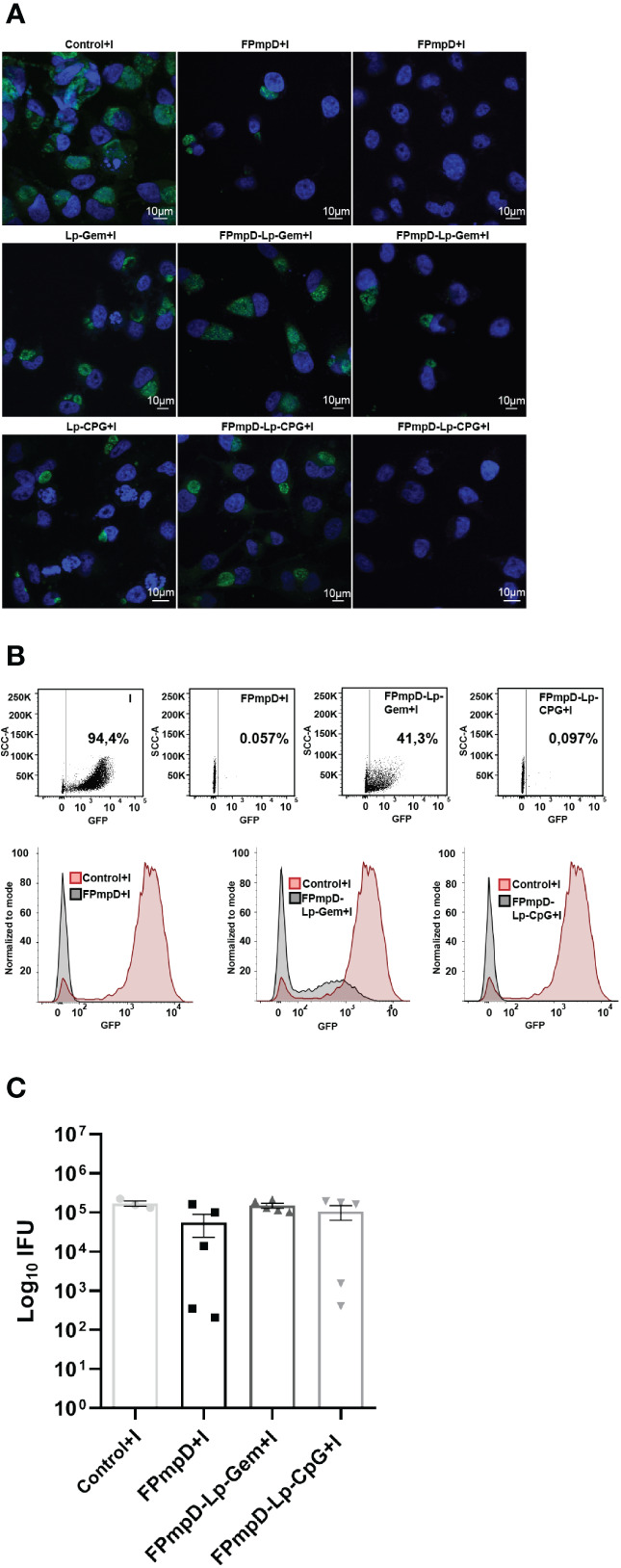
Vaginal Chlamydia shedding. HeLa cells were subjected to incubation with cervicovaginal secretions harvested seven days post-GFP-*Ct* challenge. These secretions originated from non-vaccinated infected (Control+I), or vaccinated infected mice (FPmpD+I, FPmpD-Lp-Gem+I and FPmpD-Lp-CpG+I). Following a 24 h incubation p.i., cell cultures were fixed. The inclusions formed due to the presence of infective bacteria within the vaginal fluids were detected by confocal microscopy **(A)** and quantified by flow cytometry **(B, C)**. In figure A, the confocal images exhibit the size of chlamydial inclusions developed as a consequence of the incubation with cervicovaginal secretions from a non-vaccinated infected mouse (Control+I, left panel) and a vaccinated infected mouse of the following groups: FPmpD+I, FPmpD-Lp-Gem+I, FPmpD-Lp-CpG+I (middle and right panels). The middle and right confocal images were selected from animals that presented the minimum and maximum values of IFUs, respectively. B) A representative dot-plot (upper panel) and corresponding histogram (bottom panel) derived from flow cytometry profiles. These profiles illustrate the normalized green fluorescence intensity indicative of GFP-*Ct* presence within HeLa cells, subsequent to incubation with cervicovaginal secretions. The source of these secretions incudes animals belonging to either the non-vaccinated infected (Control+I, red line) or FPmpD-vaccinated (FPmpD+I, FPmpD-Lp-Gem+I, FPmpD-Lp-CpG+I, black lines) groups. C) At day seven the inclusion forming units (Log10 IFU) were quantification in non-vaccinated infected (Control+I), FPmpD-vaccinated and infected mice (FPmpD+I, FPmpD-Lp-Gem+I, and FPmpD-Lp-CpG+I). Every symbol represents an individual animal with the bars indicating the mean ± SEM of IFU of each experimental group (Kruskal-Wallis with Dunn’s multiple comparison test; p>0.05).

In order to assess the protective efficacy of the FPmpD vaccine formulations against the dissemination of *Ct*, intravaginal challenge was administered to mice using 1.5x10^5^ inclusion forming units (IFU) of GFP-*Ct*. Cervicovaginal swabs were collected seven and 14 d.p.i, and the number of IFU was quantified. The reduction in IFU upon vaccination was indicative of protection. The samples obtained from the non-vaccinated mice that were challenged with *Ct* (Control+I) displayed a substantial presence of numerous and large chlamydial inclusions following cultivation on HeLa cells (**Figure 4A**, left panel). In contrast, some samples derived from the vaccinated mice generated some inclusions of smaller size, while others barely displayed inclusions when placed on HeLa cells (**Figure 4A**). There was a remarkablereduction in infectivity of the vaginal washes (expressed as Log_10_ of IFU, mean ± SEM) of the FPmpD+I group (55912 ± 32898) compared to the non-vaccinated mice (Control+I) (169760 ± 27603) (67% reduction) (**Figure 4C**). In contrast, the vaccinated groups with adjuvants only displayed slightly lower bacterial burden (FPmpD-Lp-Gem: 150384 ± 21673 and FPmpD-Lp-CpG:106417 ± 43352) than in the non-vaccinated group (Control+I) (11% and 37% reduction, respectively). On day 14 p.i., the IFUs shedding was undetectable even in the non-vaccinated infected mice, suggesting a natural resolution of the infection (data not shown). Therefore, among the three vaccine formulations evaluated, the FPmpD vaccine without adjuvants showed the best performance in increasing chlamydial clearance and reducing the bacterial shedding, thus achieving better control of the *Ct* infection.

### 3.4 The three FPmpD vaccine formulations elicited a sustained and strong humoral immune response

**Figure 5:**
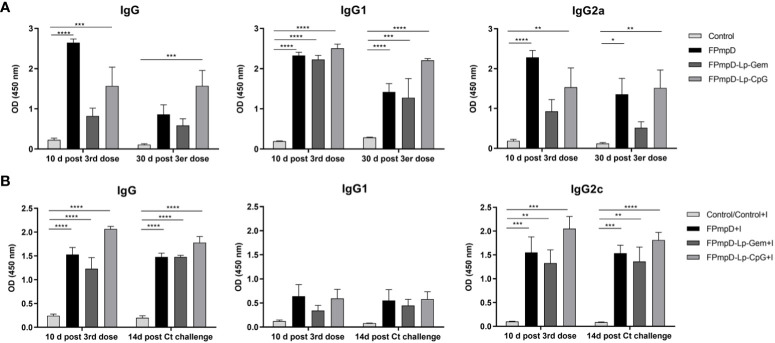
Temporal assessment of humoral immune response. **(A)** Levels of Anti-PmpD IgG, IgG_1_, IgG_2a_ antibodies in sera collected at the specified time points from non-vaccinated (Control) and FPmpD-vaccinated (FPmpD, FPmpD-Lp-Gem, and FPmpD-Lp-CpG) BALB/c mice. **(B)** Evaluation of anti-PmpD IgG, IgG_1_, IgG_2c_ antibody levels in sera acquired ten d after the final dose and 14 d post the *Ct-*challenge from non-vaccinated (Control), non-vaccinated infected (Control+I), or FPmpD-vaccinated infected (FPmpD, FPmpD-Lp-Gem, and FPmpD-Lp-CpG) C57BL/6 mice. The presented bars illustrate the mean ± SEM for each experimental group, utilizing a dilution of 1:100. The statistical analysis was performed applying a two-way ANOVA with Sidak’s multiple comparison test, signifying *p<0.05, **p<0.01, ***p<0.001, ****p<0.0001 as levels of statistical significance.

One goal of vaccination is to elicit a durable immune response against the antigen through the induction of memory cells. In this context, we examined the capability of FPmpD-based vaccine formulations to sustain the humoral response induced after the *Ct* infection. In vaccinated mice, although a minor decline in antibody levels was evident between ten days and 30 d post 3rd dose, specific IgG were detectable and remained at significantly high levels in the FPmpD-Lp-CpG group (**Figure 5A**). The three formulations induced sustained levels of IgG1, and IgG2a that continued significantly increased in the groups vaccinated with FPmpD and with FPmpD-Lp-CpG (**Figure 5A**). The group FPmpD-Lp-Gem exhibited only significantly high levels of IgG1 at 30 d post 3rd dose compared to control, non-vaccinated uninfected mice (**Figure 5A**). Subsequently, we assessed the production of anti-PmpD antibodies in the group of animals that underwent the challenge. Among FPmpD-vaccinated and infected mice (FPmpD+I; FPmpD-Lp-Gem+I; FPmpD-Lp-CpG+I), the concentrations of IgG, IgG1, and IgG2c antibodies 14 days subsequent to intravaginal Ct infection displayed similarity to those measured ten days after the third dose administration (**Figure 5B**). Conversely, the initial infection alone did not result in notable levels of anti-PmpD antibodies in the non-vaccinated infected group (Control+I), which exhibited similar antibody levels to unvaccinated uninfected mice. Consequently, vaccination with or without adjuvants induced a rapid and potent humoral immune response subsequent to the *Ct* challenge, with high levels of IgG2c likely suggesting stimulation of Th1 cells. Notably, this response was substantially more elevated than the immune response initiated by the primary infection alone (**Figure 5B**). These results show that the FPmpD formulations assayed in this study are able to elicit specific antibodies, lasting at least 30 d after the last dose. Moreover, no differences were observed among formulations, suggesting that the prime-boost strategy with free rFPmpD does not need adjuvants or immunostimulants to induce a strong humoral immune response.

### 3.5 FPmpD vaccine formulation without adjuvants prevented inflammation and genital tract pathology

**Figure 6:**
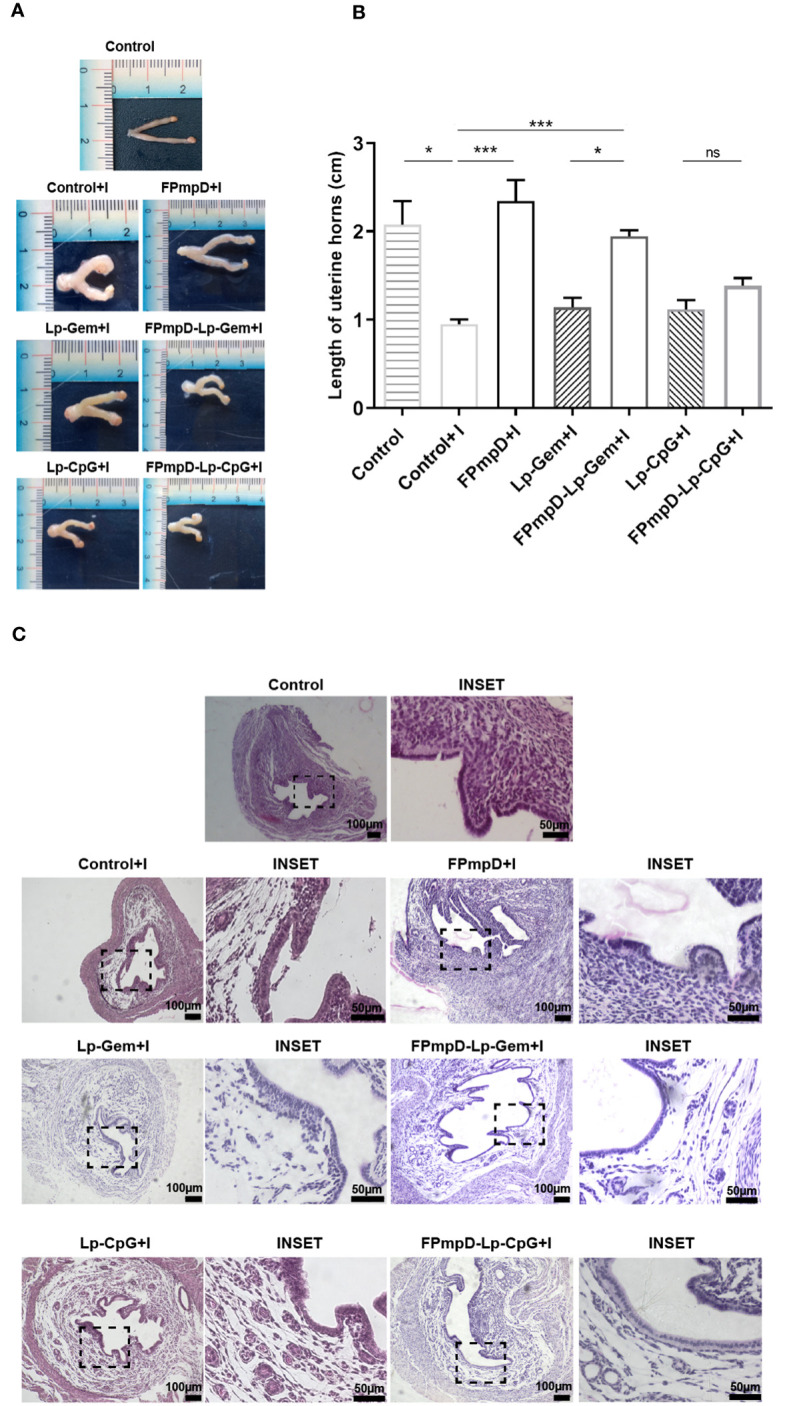
Morphological and Histological examination of uterine horns. **(A)** Illustrative depictions of uteri derived from non-vaccinated uninfected (Control) and non-vaccinated infected (Control+I) C57BL/6 mice; uteri from infected mice previously inoculated with Lp-Gem (Lp-Gem+I) and Lp-CpG (Lp-CpG+I) adjuvants; and uteri from infected mice vaccinated with FPmpD without adjuvants (FPmpD+I) and with adjuvants (FPmpD-Lp-Gem+I and FPmpD-Lp-CpG+I). **(B)** Length of uterine horns of each experimental group. The presented bars depict the mean ± SEM for each experimental group analyzed using Kruskal-Wallis with Dunn’s multiple comparison test (*p <0.05; ***p<0.001; ns, non-significant). **(C)** Representative hematoxylin-eosin-stained horn sections from uninfected non-vaccinated (Control), non- infected vaccinated (Control+I), FPmpD-vaccinated without adjuvants (FPmpD+I) and with adjuvants (FPmpD-Lp-Gem+I and FPmpD-Lp-CpG+I) infected mice on 14 d post-*Ct* challenge. The right panels of each group are magnifications of the insets of the selected areas of the corresponding images.

Considering the importance of inducing a balanced local response against infection, we studied the impact of chlamydial infection on the genital tract of vaccinated mice. As described in Materials and Methods, the genital tract of each mouse was dissected 14 d following the *Ct* challenge, enabling the evaluation of morphological and histological aspects. Representative images of uteri from the experimental groups are presented in **Figure 6A**. A recognized morphological alteration stemming from *Ct* infection is the reduction in the length of the uterine horns (34). In line with this, the infection with *Ct* led to a substantial shortening of uterine horns in the non-vaccinated mice (Control+I) in contrast to non-vaccinated and uninfected mice (Control) or the vaccinated infected FPmpD+I and FPmpD-Lp-Gem+I mice (**Figure 6B**). As expected, we detected the reduction in the length of uterine horns of Lp-Gem+I in comparison toFPmpD-Lp-Gem+I mice, attributing the protective effect to the recombinant antigen FPmpD. Surprisingly, we observed a shortening of uterine horns of mice from both the FPmpD-Lp-CpG+I and Lp-CpG groups, suggesting that the CpG-containing adjuvant induced by itself a shortening of uterine horns (**Figure 6B**). As it is shown in the representative images, *Ct* infection produces a series of changes in the tissue architecture (**Figure 6C**). The uteri of Control+I, Lp-Gem+I, Lp-CpG+I, FPmpD-Lp-Gem+I and FPmpD-Lp-CpG+I mice exhibited edema and tissue structure loss, while vaccination without adjuvants (FPmpD+I) mitigated the tissue damage caused by the *Ct* infection. Then, our results suggest that the formulations containing adjuvants (FPmpD-Lp-Gem+I, FPmpD-Lp-CpG+I) were not able to prevent the morphological and histopathological changes in the genital tract linked to *Ct* infection whereas the FPmpD-based vaccine without adjuvants efficiently reduced the chlamydial infection development in the uterine tissue and congruently decreased *Ct*-induced damage of the genital tract.

### 3.6 Lp-Gem and Lp-CpG increased TNFα expression and macrophage recruitment to the inflamed uterine tissue

**Figure 7:**
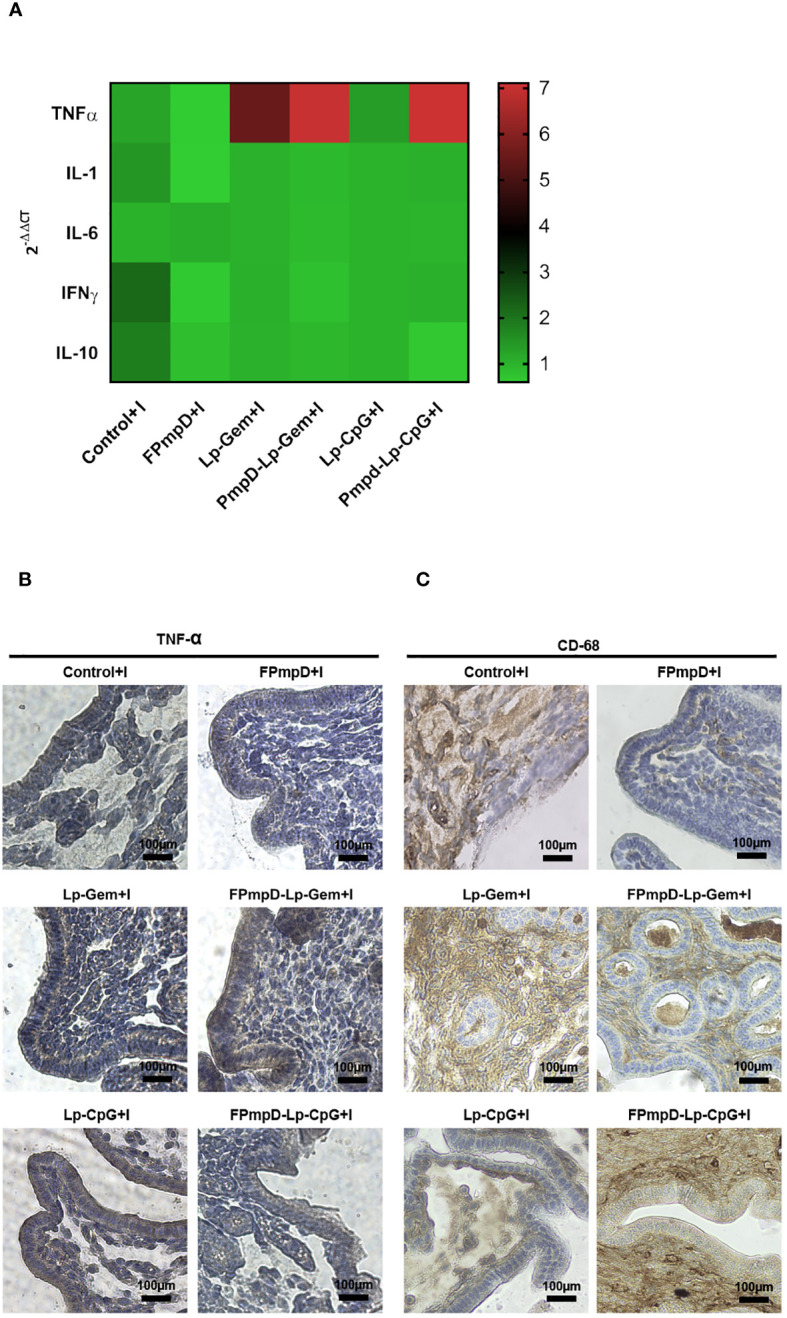
Effect on the inflammatory response of different PmpD-based formulations after the *Ct* challenge. **(A)** Cytokine mRNA expression in the uterus of C57BL/6 mice 14 days post-*Ct* challenge. The outcomes are presented as the mean relative expression of each transcript mRNA from (FPmpD+I, FPmpD-Lp-Gem+I, FPmpD-Lp-CpG+I, Lp-Gem+I, Lp-CpG+I compared to their corresponding control groups, calculated by the -2^ΔΔCT^ method. mRNA levels were normalized against GAPDH. **(B, C)** Representative immunohistochemical staining of TNFα **(B)** and CD68 **(C)** within horn sections originating from non-vaccinated (Control+I), FPmpD-vaccinated (FPmpD+I, FPmpD-Lp-Gem+I, FPmpD-Lp-CpG +I), and adjuvant treated mice (Lp-Gem+I, Lp-CpG+I) 14 d post-*Ct* infection.

*Ct* infection is known to produce inflammatory damage to the genital tract (33). Therefore, we evaluated the post-challenge inflammatory response. Given their prominent role as immune response modulators, relative expression of TNFα, IL-1β, IL-6, INF-γ, and IL-10 were evaluated in the uterine tissue 14 d post-*Ct* challenge. **Figure 7A** shows the TNFα mRNA relative expression (RE) in mice vaccinated with the adjuvanted formulations (FPmpD-Lp-Gem+I RE:6.99 and FPmpD-Lp-CpG+I RE: 7.11), displaying significantly higher values than in mice vaccinated with free FPmpD (FPmpD+I RE: 0.60) (**p<0.01) and unvaccinated infected mice (Control+I RE: 1.23) (*p<0.05). Regarding the other cytokines evaluated, a decrease in RE was observed in mice vaccinated with free FPmpD (FPmpD+I) compared to the other groups (FPmpD-Lp-Gem+I, FPmpD-Lp-CpG+I, Lp-Gem and Lp-CpG), mainly with Control+ I, although not statistically significant. These results suggest that the free FPmpD-vaccinated group (FPmpD+I) would beneficially regulate the levels of cytokines 14 d post-*Ct*-infection. Macrophages are major inducers of the proinflammatory cytokine TNFα which, in turn, has been proposed to underpin the immunopathological response triggered by *Chlamydia*. Therefore, immunohistochemical staining for TNFα and macrophages (CD68 marker) in horn sections from mice on day 14 post-*Ct* infection was evaluated. We detected increased TNFα protein expression mainly in the groups exposed to the adjuvants (FPmpD-Lp-Gem+I and FPmpD-Lp-CpG+I, Lp-Gem, and Lp-CpG) in both vaccinated and unvaccinated mice (**Figure 7B**). It is noteworthy that the main localization of TNFα protein is observed in the epithelial cells of the single-layer epithelium and the glands. We detected higher levels of macrophages infiltrating the endometrial stroma by CD68 staining in mice immunized with the adjuvanted formulations (FPmpD-Lp-Gem+I and FPmpD-Lp-CpG+I) compared to the FPmpD-vaccinated mice (FPmpD+I) (**Figure 7B**). Noticeably, FPmpD+I group presents a minor proportion of CD68 labeling compared to the rest of the experimental groups (**Figure 7C**). Mice exposed to adjuvants alone (Lp-Gem+I and Lp-CpG+I) displayed greater number of CD68 labeled cells than mice challenged with *Ct* (Control+I) (**Figure 7C**). These results are in agreement with the levels of TNFα mRNA (**Figure 7A**), allowing us to infer a relationship between a macrophage/TNFα response and the histopathological damage previously observed in the same experimental groups (**Figure 6**).

## 4 Discussion

Vaccines exemplify an achievement in the realm of public health throughout the preceding century (35). Numerous researchers have undertaken research aimed at developing a preventive vaccine against *Ct*. The main challenge is to generate a vaccine capable of triggering protective immunity without inciting undesirable immunopathological reactions within the host (33, 36). In our prior research, we assessed a vaccine strategy employing a PmpD fragment, and demonstrated the immunogenicity of FPmpD across two strains of mice. This was achieved through a prime-boost regimen involving DNA administration followed by two boosters with the recombinant protein (25). Adjuvants can improve subunit vaccine efficacy as they might enhance the nature, magnitude, direction towards particular immune profiles, and durability of the specific immune response without causing toxicity themselves (37, 38). In this study, our objective was to assess the impact of various vaccine formulations based on FPmpD, both free and with two different adjuvants, against *Ct* infection.

Several studies have underscored the significance of antibodies in conferring vaccine-induced protection against *Ct* infections, resolving primary infection, and averting re-infection (39–42). Interestingly, the formulation with free PmpD elicits similar antibody levels compared to formulations using PmpD with adjuvants. All three formulations of FPmpD vaccine administered with the prime-boost regimen elicited an increment in serum-specific antibodies after successive immunizations becoming significant following the administration of the second dose. Subsequent to the third dose, all the vaccine formulations generated a further significant increase in the antibody levels, resulting in elevated titers compared to the control condition. However, there was no significant difference in serum antibody levels among free FPmpD and FPmpD with both adjuvants (FPmpD-Lp-CpG, FPmpD-Lp-Gem). These results suggest that the adjuvants evaluated here for the administration of the rFPmpD boost would not be able to improve the magnitude of the humoral immune response generated by the free PmpD.

Unmethylated CpG motifs have immunostimulatory ability, generating high antibody levels and a cellular immune response directed towards a Th1 profile (16, 17). Given that an effective and robust immune reaction against *Ct* has been associated with neutralizing antibodies and a Th1 profile (33), this immunostimulant could beneficially modulate the immune profile elicited by FPmpD. Several authors have described the triggering of elevated levels of specific IgG2a with different vaccine formulations against Chlamydia spp (43–45), and have associated this isotype with protection against chlamydial infection (46–48). On the other hand, *gemini* lipopeptides have been extensively evaluated as transfection agents, but little is known about their immunostimulant ability. All three formulations assayed in this study elicited high IgG1 and IgG2a/c antibodies levels in both mouse strains, suggesting a balanced Th profile. The formulation containing CpG as an adjuvant triggered a higher title of specific IgG2a/c antibodies than the one with *gemini* lipopeptides. However, in the present work, adjuvants incorporation did not significantly increase the IgG2a/c titles compared with immunization with PmpD alone.

Mucosal IgA and IgG isotype represent an important contribution to immune protection against genital *Ct* infection (49–51). The contribution of vaginal IgA to mucosal defense against *Ct* remains uncertain (52). Furthermore, several authors have highlighted the role of mucosal IgG, in the absence of IgA, in protection against *Ct* (52–54). Given the significance of mucosal antibodies, the vaccination strategy evaluated in this study included intranasal administration of the protein boost. We observed a significant IgG mucosal response, with low IgA levels in cervicovaginal washings from mice vaccinated with FPmpD, both free and combined with Lp-CpG (FPmpD-Lp-CpG), in both mouse strains. Lp-Gem was not efficient in inducing a mucosal immune response. In line with our findings, other researchers have indicated that CpG ODNs elicited an IgG mucosal immune response (14, 55). Tengvall et al. demonstrated that a Herpes simplex virus (HSV-2) recombinant protein vaccine with ODN-CpG rapidly induced robust mucosal IgG and systemic Th1 responses, that conferred protection against lethal HSV-2 infection (55). The capacity of the free FPmpD vaccine to elicit mucosal IgG is remarkable and is comparable to the formulation with adjuvants that exhibited a good performance (FPmpD-Lp-CpG).

A distinctive feature of vaccination is the stimulation of memory cells that enable long-lasting immune protection. Hence, we assessed the capacity of distinct FPmpD-based vaccine formulations to sustain the generation of specific antibodies in unchallenged and *Ct-*challenged mice. The results indicate that the three FPmpD formulations tested in this study are capable of inducing a robust production of specific antibodies in sera, lasting for at least 30 d after the last dose, and exhibiting a rapid increase in antibody levels after the *Ct* challenge. These levels were substantially higher than those triggered by the primary infection itself. CpG-based adjuvants have demonstrated the ability to improve the duration of the immune response in mice vaccinated with AVA (Anthrax Vaccine Adsorbed, the licensed human anthrax vaccine (14, 56), and in calves immunized with an experimental vaccine against *Staphylococcus aureus* (57). However, when we compared the three formulations evaluated in this study, similar IgG levels and kinetics were observed, suggesting that the use of free FPmpD in a vaccine formulation would be sufficient to induce a prolonged humoral response. A similar outcome has been observed with unadjuvanted recombinant *Ct* MOMP and PmpD, which induced comparable humoral responses to adjuvanted recombinant proteins, correlating with protection (39, 58). Taking into account the high frequency of *Ct* reinfection, these results are encouraging since one of the goals of vaccination is to elicit a durable immune response capable of averting not only primary infection but also subsequent ones (31). Indeed, studies in mice indicate that the humoral immune response can play a role in clearing pathogens during a subsequent infection (6, 41, 59).

Once the capacity of the vaccinal formulations to induce an immune response was demonstrated, we assessed their efficacy in protecting against *Ct* infection. Therefore, we investigated the impact of the FPmpD-based vaccines on the shedding of *Ct* into cervicovaginal fluids and the infectivity of these samples after experimental infection. Vaginal *Ct* shedding after challenge was substantially reduced by the free FPmpD vaccine, one of five mice reduced the infectious progeny by an order of magnitude (from 10^5^ to 10^4^) while two mice reduced it by three orders (from 10^5^ to 10^2^) compared to unvaccinated infected mice. This remarkable reduction in infectious progeny was not improved by any of the adjuvanted formulations. Interestingly, the groups exhibiting post-challenge lower infectious progeny (free FPmpD and FPmpD-Lp-CpG) are those displaying higher levels of mucosal IgG. Our findings align with other publications that highlight the significance of mucosal IgG in protection against *Ct* infection (52–54).

A successful vaccine must induce a protective immune response that prevents infection while avoiding the immunopathological consequences of infection. Considering that a strong mucosal response could potentially affect fertility we evaluated the impact of vaccination on the fertility of *Ct* unchallenged BALB/c mice. Our results showed that the prime-boost strategy with the three FPmpD vaccine formulations tested did not alter fertility in this mouse strain. We faced limitations from the Institutional Ethical Committee of Laboratory Animal Care and Use related to the number of individuals required to evaluate all variables in both mice strains. We decided to assess possible fertility complications associated with the three vaccine formulations only in BALB/c immunized mice, as this strain is known to have low reproductive performance [2], providing a more challenging setting. However, it would have been interesting to analyze fertility complications associated with vaccination in C57BL/6 mice, which display strong cellular immune responses and inflammation. Besides, we evaluated fertility potential in non-infected and vaccinated BALB/c mice only. In future trials, we will analyze the putative effect of the vaccine formulations in infected and vaccinated mice, considering the probable setting of the vaccine use.

It is noteworthy that adjuvants are designed to enhance the immune response, but this is associated with a risk of adverse reactions (3, 60). Given that the pathogenesis of chlamydial disease is partly immune-mediated due to inflammatory reactions in the genital tract (3, 60), we evaluated the histological and morphological features of the uterine horns in vaccinated and *Ct*-challenged mice. We observed that the free FPmpD vaccine formulation did not cause damage to uterine horns, confirming our previous finding (25). Furthermore, FPmpD vaccine prevented *Ct*-induced uterine damage after chlamydial vaginal challenge. In contrast, mice vaccinated with the adjuvanted vaccine formulations (FPmpD-Lp-Gem+I, FPmpD-Lp-CpG+I) displayed an enhanced inflammatory response after exposure to *Ct*, likely associated to immunopathological tissue damage. Although some clinical trials suggest that ODN-CpG are generally safe and CpG-adjuvanted vaccines have similar safety profiles (61, 62), other reports highlight a rise in the severity and/or frequency of local adverse events and systemic symptoms with CpG-adjuvanted vaccines (14, 63). Studies with animal models have indicated that under specific circumstances, CpG motifs can facilitate the development of harmful autoimmune reactions. For example, the administration of CpG DNA with clamidial antigen has been shown to induce autoimmune myocarditis (64) or arthritis (65). Regarding *gemini* lipopeptide, limited information regarding its adverse effects is available, but our findings suggest the triggering of an inflammatory response. Notwithstanding, its ability to induce high antibody levels makes this molecule a potential immunostimulant that may be useful for other vaccines and deserves further investigation.

The pathology associated with *Ct* infection stems from localized tissue damage, primarily affecting epithelial cells as a result of the infection itself. Subsequent detrimental host inflammatory responses lead to permanent scarring (3, 66). Several studies with *C. trachomatis* and *C. muridarum* have demonstrated the migration of macrophages to sites of chlamydial infection. These macrophages engage in the phagocytosis of bacteria and subsequently release pro-inflammatory cytokines (41, 67–69). Two predominant cytokines typically generated during inflammation are IL-1 and TNFα, which activate polymorphonuclear leukocytes, stimulate the production of prostaglandins and collagen, increase integrin expression, and the secretion of IL-8, and IL-6, among others (70, 71). In this research, we assessed the post-challenge inflammatory response by analyzing the mRNA expression of TNFα, IL-1b, IL-6, INFγ, and IL-10 in uterine tissues 14 d after *Ct* challenge. Mice receiving adjuvanted formulations demonstrated a notable rise in TNFα expression. This response may contribute to infection-induced tissue damage. Furthermore, we found that epithelial cells of uterine tissue are the main producers of TNFα. However, we cannot exclude the possibility that macrophages also contribute to the release of this proinflammatory cytokine, supported by the observation of macrophages in the uterine tissue 14 d after *Ct* challenge, particularly in vaccinated mice with adjuvants. These mice also showed damage and destruction of cells in the uterine tissue. The inflammation subsequent to chlamydial infection may induce apoptosis (66). Then, we could speculate that the death of cells may be due to apoptosis induced by the inflammatory immune response associated with macrophages and TNFα.

In summary, we demonstrate that prime-boost immunization using free FPmpD prevents chlamydial infection and damage to the reproductive tract. This strategy includes a first dose of DNA, which has its own immunostimulatory activity, partially due to CpG motifs that can direct the immune response directed toward the Th1 profile. While a first dose of a vaccine is crucial in determining the response profile (72), the adjuvant function of DNA itself could explain the lack of improvement of the immune response in the Lp+CpG adjuvanted FPmpD formulation. On the other hand, a boost with rFPmpD alone is enough to generate a persistent humoral immune response without triggering inflammation after the *Ct* challenge, which correlates with protection against uterine tissue damage. An ideal vaccine must be feasible to produce, and the formulation containing free FPmpD meets this requirement as it has fewer components, implying lower risk and a better cost-benefit ratio. For this particular infection, the inflammatory non-specific response mediated by Lp-CpG and Lp-Gem adjuvants could have counter-productive effects from an immunopathological standpoint. In agreement with other authors (36, 73, 74) we have observed that adjuvants can also induce adverse events, such as an over-amplified immune response, which can cause immunopathological damage to the host genital tract, and does not contribute to chlamydial clearance. In summary, our findings support that the best vaccine formulation comprises DNA prime-free rFPmpD boost. This vaccination strategy is promising for controlling sexually transmitted infections caused by *C. trachomatis*. Further studies are need to better characterize the induced cellular immune response induced and to evaluate the efficacy of the FPmpD-based vaccine in humans in the future.

## Data availability statement

The raw data supporting the conclusions of this article will be made available by the authors, without undue reservation.

## Ethics statement

The animal study was approved by Animal protocols were approved by the Institutional Advisory Committee on Research Ethics and Security (FBCB-UNL, Argentina) and by the Institutional Committee on Laboratory Animal Care and Use (CICUAL, UNCUYO, Argentina). The study was conducted in accordance with the local legislation and institutional requirements.

## Author contributions

RR: Conceptualization, Data curation, Formal Analysis, Investigation, Methodology, Validation, Visualization, Writing – original draft, Writing – review & editing. DD: Conceptualization, Formal Analysis, Investigation, Methodology, Visualization, Writing – review & editing. MA: Investigation, Methodology, Writing – review & editing. NF: Methodology, Writing – review & editing. IR: Methodology, Writing – review & editing. AL: Investigation, Methodology, Writing – review & editing. DS: Methodology, Writing – review & editing. MD: Conceptualization, Data curation, Formal Analysis, Funding acquisition, Project administration, Resources, Supervision, Validation, Visualization, Writing – original draft, Writing – review & editing. CV: Conceptualization, Data curation, Formal Analysis, Funding acquisition, Project administration, Resources, Supervision, Validation, Visualization, Writing – original draft, Writing – review & editing.
